# Motivating playgrounds: understanding how school playgrounds support autonomy, competence, and relatedness of tweens

**DOI:** 10.1080/17482631.2022.2096085

**Published:** 2022-07-03

**Authors:** Thea Toft Amholt, Birgitte Westerskov Dalgas, Jenny Veitch, Nikos Ntoumanis, Jeanette Fich Jespersen, Jasper Schipperijn, Charlotte Pawlowski

**Affiliations:** a Active Living, Department of Sports Science and Clinical Biomechanics, University of Southern Denmark, Campusvej 55, 5230, Odense, Denmark; b KOMPAN A/S, C.F, Tietgens Boulevard 32C, 5220, Odense, Denmark; c Deakin University, Geelong, Australia, Institute for Physical Activity and Nutrition (IPAN), School of Exercise and Nutrition Sciences, Melbourne, Australia; d Danish Centre for Motivation and Behaviour Science, Department of Sports Science and Clinical Biomechanics, University of Southern Denmark, Campusvej 55, 5230, Odense, Denmark

**Keywords:** Tweens, school playgrounds, psychological needs, self-determination theory, physical activity, health

## Abstract

**Purpose:**

Physical activity (PA) is an important factor contributing to general health. PA declines rapidly during tween years (9–12 years) when children’s social world changes. School playgrounds can contribute substantially to children’s PA, but little is known about how to motivate tweens to use school playgrounds. Using the three basic psychological needs (autonomy, competence, and relatedness) proposed by the Self-Determination Theory, this qualitative study aimed to investigate how school playgrounds can support tweens’ needs.

**Methods:**

Tweens (n = 56) participated in focus group go-along interviews in their school playground.

**Results:**

We found that tweens needed a variety of play possibilities (autonomy) that challenged their skills (competence) as well as areas to retract and be with friends (relatedness).

**Conclusion:**

This research highlights the importance of incorporating tweens’ perspectives in playground design to attract and retain them in play and PA in school playgrounds.

Being physically active is one of the most important factors contributing to physical and mental health during childhood (Aziz & Said, 2015; Black et al., 2015; Bundy et al., 2017). Physical activity (PA) can reduce the risk of obesity as well as chronic diseases, in particular diabetes and cardiovascular diseases (Telama et al., 2005). Despite the well-known benefits of PA, overall PA levels are declining with more than 80% of children aged 5–17 globally failing to meet the PA recommendations of the World Health Organization (Jones et al., 2013). The rate of decline in PA is especially high during the tween years (9–12 years) (Rauner et al., 2015). In this period, children’s social emotional world changes and their focus on social status and relationships with peers increases thus resulting in a shift of priorities lowering PA (Frost et al., 2012; Lightfoot et al., 2009). Furthermore, their behaviour changes from intuitive to more self-conscious which leads to an ongoing evaluation of their own appearance to others (Lightfoot et al., 2009). These changes call for a specific need to understand how to motivate tweens to be more physically active.

A substantial amount of children’s waking hours are spent at school; research has shown that up to 40% of children’s daily PA can be obtained during recess periods (Ridgers et al., 2006). Therefore, the built environment at school and especially school playgrounds have received increasing empirical attention. Studies have shown that well-equipped playgrounds can enhance children’s PA (Ross & Francis, 2016). One study using direct observations concluded that a variety of play equipment could enhance children’s moderate-to-vigorous PA (Cohen et al., 2020), and another observation study concluded that colours and features increase playground use (Czalczynska-Podolska, 2014). Furthermore, an interview study of tweens’ health behaviour showed that the opportunities provided by the built environment influenced their perceived motivation (Tay et al., 2021). One study using GPS and accelerometry to map tweens’ PA patterns in school playgrounds concluded that the most active school playgrounds included ball game areas, areas away from main playgrounds, and climbing equipment (Amholt et al., in review). Another study using structured observations to investigate different types of tweens’ play in school playgrounds concluded that tweens spend 80% of their time on either physical or talkative play including physical games, competitive play, and hanging out with friends (Amholt et al., 2022). This is in contrast to younger children, who prefer constructional and symbolic play such as play building with sand and imaginative play e.g., playing pirates or hospital (Dyment & O’Connell, 2013). These studies concluded that the play preferences of tweens differ from those of younger children and should be considered when designing school playgrounds to attract and motivate this age group to be more physically active.

Despite the well-known benefits of school playgrounds and the developmental changes happening during tween years, limited research has been conducted on motivational factors for tweens’ school playground use. Previous studies call for direct child involvement in playground research (Pawlowski et al., 2019; Veitch et al., 2006, 2007). To better understand how school playgrounds can facilitate use by tweens, a theoretical concept is needed. According to the social ecological model, there are several factors that affect behaviour (Bronfenbrenner, 1994). These factors include personal as well as contextual levels that interact to shape our behaviour (Flôres et al., 2019). The different levels in the model also imply that the environment is not only physical but also interact with the perceived environment as explained by the theory of affordances (Kyttä, 2002). Focusing on the perceived possibilities that the built environment provides, health researchers have investigated motivation for health behaviour. Application of motivational theories in health and PA research has increased substantially in the last 20 years (Hancox et al., 2018). Many health researchers have used the Self-Determination Theory (SDT) (Deci & Ryan, 2017) to conceptualize and explore motivation (Ng et al., 2012; Ntoumanis et al., 2021). Systematic reviews and meta-analyses have concluded that the SDT is a viable conceptual framework for understanding the relationship between motivation, health-related behaviour, and health outcomes (e.g., Ntoumanis et al., 2021).

## Self-determination theory

The SDT distinguishes three basic psychological needs that should be satisfied for individuals to feel autonomously motivated (Deci & Ryan, 2004). In other words, motivated by enjoyment and personal value of the activity. The first need, autonomy, is the experience that choices and actions are self-endorsed and self-initiated (Deci & Ryan, 2017). The second basic need, competence, is the ability to accomplish important personal goals and experience task mastery (Deci & Ryan, 2017). The third basic need, relatedness, is the experience of warmth, support, and caring from and for other people (Deci & Ryan, 2017). These basic needs can either be supported or thwarted by the context and social interactions which an individual (e.g., a tween) experiences (Deci & Ryan, 2017). The support of the three basic psychological needs can be achieved via important social agents (e.g., parents or teachers), but what is relevant for this study is that it can also be achieved via appropriately constructed physical contexts such as built environments (Deci & Ryan, 2017). The majority of the research conducted on need support has been interested in interpersonal relationships. whereas the research on need support provided by the built environment is limited (Hancox et al., 2018). For instance, in the context of primary school education, intervention studies have been conducted to train teachers to deliver lessons in need-supportive ways. Such interventions have shown benefits in terms of motivation for physical activity as well as health-enhancing levels of physical activity (e.g., Escriva-Boulley et al., 2018; Lonsdale et al., 2019).

To support autonomy, built environments should facilitate options and possibilities to enable children to actively decide for themselves and most importantly, such environments should encourage choice (Vansteenkiste et al., 2005). To support competence, built environments should facilitate possibilities for developing skills and experiencing success and mastery (Deci & Ryan, 2017). The environment should act as a structure for activities that provide optimal task challenges and useful feedback, and children should experience how to overcome barriers. Less research has been conducted about the support of relatedness via built environments. One major consideration, though, is the facilitation of opportunities for children to be together (Deci & Ryan, 2017). For instance, one study suggested that built environments should promote teamwork and cooperation (Leyton et al., 2017). These studies thus indicate the potential of contexts such as built environments to provide support for autonomy, competence, and relatedness. Hence, built environments such as school playgrounds that support the three basic needs will enhance tweens’ autonomous motivation for using the playground. Therefore, we need research focusing on the potential of school playgrounds to support healthy, social, and physical activities via fostering psychological need support. This information will provide important knowledge on how to take further advantage of school playgrounds as a health promoting context. On this basis, the aim of this study was to understand how playgrounds can support the need for autonomy, competence, and relatedness of tweens to increase their autonomous motivation to use playgrounds.

## Methods

### Study design

This study was designed as a descriptive study (Darbyshire et al., 2005). The study has a deductive approach drawing on the SDT framework to understand how school playgrounds facilitate support of the three basic needs that result in autonomous motivation of tweens. Using a qualitative approach, we conducted semi-structured focus group go-along interviews and analysed the transcriptions using the three concepts from the SDT; autonomy, competence, and relatedness.

### Sampling

Well-equipped school playgrounds with play equipment targeting tweens were sampled. The sampling was conducted in cooperation with the largest Danish playground company, KOMPAN. As no common database of playgrounds in Denmark exists, this cooperation provided us with a unique possibility for conducting a strict sampling procedure. KOMPAN has a market share of approximately 35% of playgrounds in Denmark and provided us with a database of approximately 4000 playground orders within the last five years. Playground selection criteria included: 1) outdoors, 2) had a minimum of five playground pieces, 3) had play equipment relevant for 9–12-year-olds, 4) was built within the last five years (i.e., finished between January 2015 and December 2019), 5) was located at schools, and 6) a minimum cost of approximately 50.000 USD (300.000 DKK) to ensure they were well-equipped. This resulted in 31 school playgrounds of which we selected seven that differed in factors known to influence children’s PA including playground size, number of children attending the school, variation of urban and rural school settings, and variation in play equipment.

### Participants

Invitations to participate were sent to the school principals of the seven selected schools. Four schools accepted our invitation, two did not respond, and one did not wish to participate. Information letters were sent to school principals and teachers from the four interested schools, and the PI held a meeting with one primary contact person at each school. Invitation letters for a child to participate in the study were forwarded to parents of children in grade 4 through 6 (9–12-year-old children). On behalf of their child, parents could indicate if they were interested in participating using a link provided on the letter. All parents who indicated interest received an informed consent agreement to sign. A total of ten groups of 5–6 tweens from four schools participated in the study; 56 children in total (33 boys (59%) and 23 girls). From school one and two, three groups from grade 4, 5, and 6 participated. From school three, two groups from grade 4 and 5 participated, and from school four, two groups from grade 5 and 6 participated. The exclusion of the grade 4 at one school and grade 6 at another school was due to these grades not having access to the school playground. Students were randomly selected to participate if the number of children from each grade level at each school that were allowed to participate was greater than six.

### Procedure

Focus group go-along interviews were selected for this qualitative study as they are effective in gathering data on child perspectives (Hayball & Pawlowski, 2018). They also create interaction-based conversations where children help each other verbalize their attitudes, memories, and statements (Adler et al., 2019; Darbyshire et al., 2005; Horner, 2000; Krueger, 2014). The go-along interview has the advantage of combining live interviews with observation (Kusenbach, 2003). We used a semi-structured interview guide with predefined questions—and follow up questions—that emerged as relevant during the interview. The interviewer was a trained researcher with extensive interview experience. The interview guide was developed by operationalizing the three basic psychological needs proposed by the SDT with questions aimed to prompt responses to the three needs in tweens’ use of school playgrounds. For example, questions included: “Who decides what to play?” (autonomy), “Is there something about this activity that is difficult?” (competence), and “Do you play here alone or with others?” (relatedness). The procedure and interview questions were pilot tested on one group of three children prior to data collection. One interviewer conducted all interviews that consisted of two phases (see Table I). Phase 1 was completed in the classroom and involved an introduction to the purpose of the study and a series of questions aimed at familiarizing the interviewer and the children. In phase 2, each group completed a focus group go-along interview around the school playground. The interview questions are outlined in Table I. Interviews were recorded using an audio recorder as well as a small camera for video recording. Video material was used to identify the location on the playground when children referred to memories or feelings related to specific places or equipment. Interviews lasted approximately 45 minutes for each group and were completed in August-September 2021.

**Table I.** Interview procedure.

Phase	Activity
1.Open focus group discussions in classroom	Interviewer facilitated informal conversation. Questions focused on favourite play activities:
	Tell me about your school playground What are your favourite play activities? Do you do this activity alone or with others? Why do you like this activity? Who decides that you should do this activity? Is there something about this activity that is difficult?
2. Go-along interview in playground	Questions focused on their favourite play areas:
	What are your favourite play areas? Do others like this area too? Why do you like this area? Do you play here alone or with others? Who decides that you should play here? Is there something difficult about this area?

### Data analysis

The data were analysed using the seven steps from the Framework Method (Gale et al., 2013). In step 1, *transcription*, the recordings were transcribed and anonymized immediately after each interview by the same researcher that conducted the interviews. Transcriptions from the interviews were entered into the NVivo software (version 12) for coding, categorizing, and summarizing data. In step 2 and 3, *familiarization and coding*, data were analysed independently by two authors who familiarized themselves with the interview data by reading the interviews and coding the data in themes corresponding with the three basic psychological needs of autonomy, competence, and relatedness. In step 4, *development of theory*, the analysed data were described for each of the three basic psychological needs according to how the school playgrounds supported or thwarted these three basic needs. In step 5, *application of theoretical framework*, the final descriptions of the coding were used to go over all ten interviews again applying the categories and descriptions developed in step 4. To maximize inter-reliability, we compared the two authors’ coding and discussed discrepancies until consensus was reached. Inter-coder reliability was calculated using Cohen’s Kappa (Howell, 2016) and revealed a coefficient of 0,71 which is considered very high. A comparison of the coded sections of the transcriptions of coder 1 and 2 revealed an agreement percentage between 95,72% and 99,98% on character level. In step 6 and 7, *charting and interpreting*, the analyzed interviews and final codes were collected in matrixes, read, and described by the two coding authors. These descriptions are presented in the results section.

## Ethics

The Research Ethics Committee at the University of Southern Denmark (20/29,520) and Legal Services, University of Southern Denmark (11.068) approved the study. Only children with both parental consent and children’s oral assent participated. Parents and children approved the use of as audio and video recording. Children and parents were informed that it was voluntary to participate and that they could withdraw from the study at any time.

## Results

**Figure 1.**
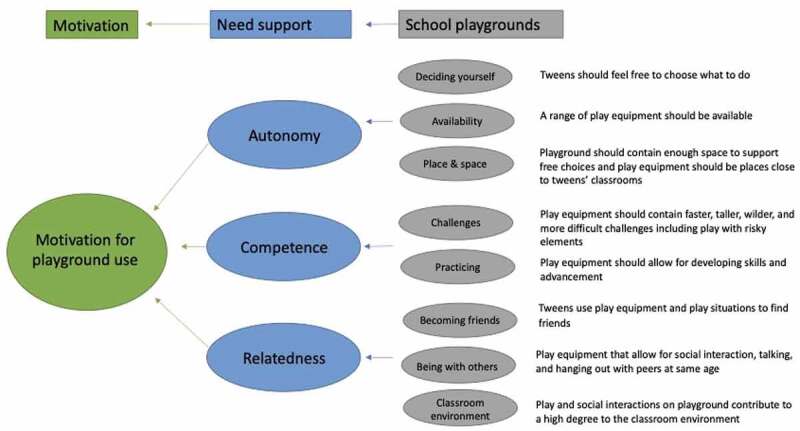
Overview of results. Need support of tweens on school playgrounds.

From the analysis, several features of the school playgrounds that either supported or thwarted autonomy, competence, and relatedness emerged. An overview of the findings can be found in Figure 1.

### Autonomy

In our analysis, we found that the school playground supported autonomy when the tweens could decide for themselves what to do. This was achieved by the availability of play equipment and by the space available at the playground. The tweens described the school playground as a place where they could easily decide what they wanted to do and that this differed from the classroom where their teacher was in control. They described that it was easy to join in new games and choose to play in different areas on the school playground.

*”Then maybe others will pick something different. And others something different. If you think something is funny, that you want to join, then you should just go over there and ask if you can join”* (girl, 9 years, school 3).

Often, a game became popular in a specific period and the children unanimously agreed what to do during recess. Sometimes larger games were decided by vote count. What inhibited autonomy was when one specific child decided what everyone else should play. This happened e.g., both at a specific piece of play equipment that was popular and when the class possessed only one ball.

*“Sometimes it [the game decided] depends on who gets the ball. If we want to play dodgeball, it is the person who gets the ball first that can decide if we can play that or not … ”* (girl, 10 years, school 3).

Variety was also identified as key for the school playground support of autonomy. If a specific game (such as football or a tag game) was played too much or a piece of play equipment was used for a long period, the children expressed a need for change: “*I just think that when we have played something for a longer time, it becomes less nice*” (girls, 12 years, school 1). To support autonomy on school playgrounds, the availability of a range of different play equipment pieces and play possibilities was important. Children referred to how they could pick and choose what they wanted to do, join in on activities, and skip parts of a game or an obstacle course if they wanted to. The number of play equipment pieces available was crucial for whether the children felt they had something to choose from. Furthermore, they described a need for variety in play opportunities. Being able to shift between being very physically active as well as having areas to hang out and talk was important. If the school playgrounds possessed play equipment that was unique, the tweens would preferably choose these pieces. New pieces of play equipment or pieces that were found nowhere else were considered interesting. The need for autonomy was thwarted if teachers divided the playground in areas for different classes and the children could not choose where to play. Two girls from school 2 described this:

Girl 1, 11 years: *There is also a playground up there, but we are not allowed to use it, it is mostly for 1^st^ graders.*

Girl 2, 11 years: *I find that a little annoying because they have some cool pieces.*

The playground did not support the need for autonomy if the equipment was damaged or if there were too few play opportunities. A girl said: *”But we don’t have much to do besides that we can play football and basketball. There are not many play items out here”* (girl, 10 years, school 4).

If there was not enough equipment, the children had to run fast when recess began to “take” the equipment. Some schools had to make rules about turn taking: *”It’s just that the worst part about this one is standing in queue and waiting for your turn. Then you get one try and the break is over”* (girl, 11 years, school 1). Well-placed equipment and enough space for the children on the school playground were important. Too many children on a small playground area created a feeling of being squeezed and the areas becoming noisy. Hereby, their ability to choose freely what to play and where to be, was thwarted. Furthermore, if the play equipment was located far from the classroom, the children described that it was used less frequently because it took too long to run inside when the bell rang: *“We used to play here when we were young … Now our classroom is so far away, and we must get to class in time”* (girl, 10 years, school 2).

### Competence

Factors related to tweens’ competence on school playgrounds included the challenges available on the playground, the possibility for practicing and become better, and the options for “cool-looking” risky play situations. The need for competence was supported if children could perform skills such as climbing up a tall tower or spinning faster and faster. The tweens continuously looked for challenging equipment and activities that were higher, wilder, faster, and more difficult. When asked what she wished the play equipment to be like, a girl answered: *”I would like it to be challenging to get up there. Yes, because I think it is like … A little challenging to be able to get high up”* (girl, 11 years, school 1).

The children were proud of trying and completing difficult challenges. In a conversation, two boys from school 3 described their need for challenging activities:

Boy 1, 11 years: *It would be awesome with a taller tower. Then you have a better view. And it’s also more difficult*.

Boy 2, 12 years: *For example, when the tower was new, it was like, you were pretty cool if you dared to climb up there*.

When the equipment lacked age-appropriate challenges, the children described less competence support. For example, play equipment where they could reach the ground when hanging from their arms.

Interviewer: *Isn’t it tough for the arms to use the monkey bars?*

Boy 1, 10 years: *No, not on that small one we have*.

Boy 2, 10 years: *Yes, that is why I sometimes cycle all the way to Faarevejle [town] because there is a monkey bar that is two meters tall. And I can do it*.

This was also described in risky play situations where the tweens sought big challenges. This could be spinning so fast that they got sick or climbing places that they could easily fall from. The feeling of excitement from risky play was explained by a group of children demonstrating a specific game they played during recess.

Boy, 11 years: *And that one [points to a piece of spinning equipment]. That one can make you sick (laughs). And you can get hit in the head by it. Suddenly, it comes towards you with giant speed and then right into your face*.

Interviewer*: Don’t you get afraid of using it then?*

All tweens: *NO!*

Adding an element of risk to their play activities got them excited and challenged them to new activities. This element of risk should be present but not too high. This balance was explained by a boy: *“ … Not something that is likely to go wrong. That’s not something I want to do. But it’s funny that there can be a risk, that it won’t just go as it’s supposed to”* (boy, 11 years, school 4). Furthermore, our findings revealed that activities on school playgrounds supporting competence were to practice, learn something new, and get better. The tweens slowly advanced when they used the obstacle courses or climbed the tall climbing towers. They described doing difficult things step by step and progressing with help from others. One girl explained how she practiced her climbing skills on different platform levels in a large obstacle course: *”If you are not quite able to do the tall part yet, then you can practice on the lower ones and then afterwards try the tall ones”* (girl, 9 years, school 2).

### Relatedness

For school playgrounds to support relatedness, we found that social areas on the playground were very important. They could facilitate new friendships, provide possibilities for looking cool, hanging out with friends, and create a positive social environment. Generally, the school playground was described as a positive social arena where friendships could emerge and evolve. The tweens described that it was easy to invite others to join them on play equipment or games they had already made up. Social interactions on the school playground became a way to get to know each other better. Some tweens explained how swinging, spinning, or climbing together could bring friends closer.

*“Yes, well, Sofie and I at one time … We became best friends. We spun around so much together and then we became friends. Best friends actually. And then this girl called Anna joined us and we became best friends and now we are all best friends and have been that for a long time”* (girl, 11 years, school 1).

Furthermore, social interactions on the school playground established social hierarchies. Activities that could either be cool or uncool influenced their relations with peers. A boy explained how he could use the play equipment to impress others and find friends: *“You can do something that you are good at already and then show it”* (boy, 11 years, school 3). According to the tweens, their activity preferences had changed compared to when they were younger. Age-appropriate activities with same age peers could provide more meaningful interactions, and playground areas that facilitated areas to hang out with peers of the same age supported tweens’ need for relatedness. Play equipment placed next to the classrooms of the younger children was less popular: *“When you are in 6^th^ grade, you don’t play with 4^th^ and 5^th^ graders. Then you are a little bigger. It is not that cool”* (girl, 12 years, school 1). It was described as very important to have someone to be with and that play equipment with sufficient space for social interactions made it more fun to be physically active. Hanging out, talking, and playing with others gave the tweens positive feelings.

*“Me and my friends we used to climb high up in the tower and then there are these things you can sit on, leather things, and then we sit there for the rest of the break talking”* (boy, 12 years, school 3).

The need for relatedness was discouraged, though, if a tween preferred activities that no other tweens liked and did not want to play alone. It was important for the tweens to experience relatedness to others while playing and thus, relatedness was not supported if they had no one to play with. A conversation between the interviewer and two boys from school 1 showed this:

Interviewer to boy 2: *And you skate?*

Boy 1, 12 years: *Yes, the first ten minutes, you do, and then you move on to doing something else*.

Boy 2, 12 years: *It’s because no one else likes to do it*.

Finally, the tweens described how positive experiences and incidences from their play situations were often continued when they entered the classroom after recess: *”If you did something funny during the break, you can just laugh of it inside the classroom”* (boy, 12 years, school 3). In contrast, negative experiences such as disagreements and discussions on the school playground inhibited their need for relatedness and created a negative social classroom environment: *”You have time for almost nothing and then you are angry when you get inside because you had a fight with someone and then you are really angry”* (girl, 10 years, school 4).

## Discussion

In this study, we analysed how school playgrounds support the three basic psychological needs from the SDT; autonomy, competence, and relatedness. Our sampling procedure revealed that very few playgrounds have specific facilities for tweens. We found that playgrounds could support each of these three psychological needs. School playgrounds supported the need for autonomy when the tweens could freely choose from a variety of different play equipment pieces on the playground. Furthermore, playgrounds should provide enough space to avoid noisy areas and the feeling of being squeezed together. To support competence, playgrounds should facilitate challenges at different levels, enabling the tweens to practice and experience task mastery. These challenges should contain an element of risk where the tweens could experience a high degree of competence. Finally, playgrounds provide a unique possibility for making and strengthening social bonds. To support relatedness, playgrounds should include areas to hang out and talk with friends of similar age. Including social areas on school playgrounds could therefore be considered a determinant for tweens’ need support of relatedness in school playgrounds.

### Need support on school playgrounds

The tweens expressed a need for deciding themselves what to do on the playground. Autonomy was inhibited if recess was characterized by high teacher control. This finding aligns with other health studies on autonomous motivation and need support which have shown that children are less likely to participate in sports characterized by a high degree of adult control (Baceviciene & Jankauskiene, 2021; Ryba et al., 2012). Yet, other studies have shown that teacher-initiated activities created more play across gender and age groups (Huberty et al., 2011; Pawlowski et al., 2016). These conflicting findings might indicate that different children benefit from different degrees of control when playing. Future research should aim to understand these preferences.

Space was also a consideration for autonomy. Tweens expressed that their autonomy was restricted if too many children were squeezed together in too little space. This has also been shown in other studies on need support in outdoor spaces. A previous study concluded that motivation for being active outdoors was positively associated with the amount of space provided (Islam et al., 2016). Other studies of children’s park use have shown that the variety of play equipment and opportunities for children’s play are positively associated with use and PA (Cohen et al., 2020; Sylvester et al., 2014; Veitch et al., 2021).

We also found that school playgrounds should provide challenges to support the need for competence of tweens. This aligns with research using the SDT that has stated that the built environment should facilitate opportunities to practice skills (Deci & Ryan, 2017). The tweens described playground areas for younger children as boring which lowered their competence support. Previous research has found that older children felt discouraged when their school playgrounds did not provide appropriate challenges (Kreutz et al., 2021). This emphasizes the importance of incorporating challenges at different levels in school playgrounds. Studies of tweens’ PA in school playgrounds found that ball game areas and climbing equipment were characterized by high levels of PA (Amholt et al., in review; Martínez-Andrés et al., 2017). We found that tall climbing equipment was described as challenging and that soccer fields were used to compete and practice new skills. The need for challenges also included risky elements such as spinning equipment. Previous studies within the field of risky play have stated that risk taking plays a major role in children’s use of playgrounds (Brussoni et al., 2012; Herrington & Brussoni, 2015). Here, risk is not denoted with danger but is used for a situation where a child can recognize and evaluate a challenge and decide how to handle it (Brussoni et al., 2012). A systematic review concluded that risky play was positively associated with a number of health indicators including physical play (Herrington & Brussoni, 2015). Providing opportunities for thrilling, challenging, and risky play, will motivate and engage children in physically active play. These conclusions emphasize the need for school playgrounds to encourage tweens to push their limits and develop competence.

Finally, we found that school playgrounds should include areas to hang out and be social. One major way the school playground provided support of relatedness for tweens was by including places to be with their peers. Previous health research within an SDT framework has emphasized the importance of being together to build relatedness (Leyton et al., 2017). A study on children’s view on park features also showed that the size of the play equipment was important for social interaction with larger play equipment pieces preferred for social interaction (Veitch et al., 2020). Previous research found that tweens spend one fifth of their time on playgrounds talking and hanging out which emphasizes the need for playground areas away from younger children where tweens can hang out and spend time with each other (Amholt et al., 2022). School playgrounds should include areas specifically for older children; however most playgrounds are designed for children younger (< nine years) possibly leading to an exclusion of older children (Czalczynska-Podolska, 2014; Veitch et al., 2007). A longitudinal study of motivational factors found that peer influence was often the most important factor when considering motivation in health domains (Ntoumanis et al., 2012). Being social is also especially important for girls, who often tend to be less active than boys (Laird et al., 2018). Recent studies have shown that face-to-face social interactions decrease in tween years due to the increasing time spent with mobile devices and screens (Larson et al., 2019). The negative impact of screen time on time spent outdoors reinforces the importance of ensuring that outdoor play spaces are appealing (Larson et al., 2019). Previous studies have shown that including children in the design processes of their outdoor areas enhances their use of the areas (Andersen et al., 2019; Christiansen et al., 2017; Roberts et al., 2018). On this basis, playground designers and playground owners should consider the needs of children in all developmental stages. Involving tweens in the design process would further support their autonomy.

### Strengths and limitations

The use of group based go-along interviews has great advantages for understanding children’s perceptions and experiences of their built environment (Darbyshire et al., 2005; Pawlowski et al., 2016). The go-along interview allows for understanding children’s views in-situ (i.e., at the playground) so they don’t have to recall past experiences. Giving children a voice and listening to their needs provide important knowledge that cannot be captured using device-based measures and observational tools (Hayball & Pawlowski, 2018). Previous qualitative studies on children’s perceptions of their physical environment emphasize that children appreciated the opportunity to discuss their perspectives and felt valued that researchers take time to listen to them (Agar, 1996; Hayball & Pawlowski, 2018). Other methodological approaches for participatory study designs include the use of photographs (Adams et al., 2017), photo-voice (Spencer et al., 2021), and drawings (Cooper et al., 2017). These approaches may have provided an opportunity to create further dialogue and should be considered in future research. This study has highlighted that tweens appreciate the support of autonomy. We stived to support this by having the tweens show the interviewer around their school playground. Furthermore, it is important to acknowledge that although focus group interactions can stimulate debate among children and encourage them to explore their views and create new ideas (Clark, 2009; lark, 2011), they also have the disadvantage of social desirability, implying the risk of children seeking conformity (Daley, 2013). The interviewer strived to create an honest dialogue and asked for competing opinions, however, it was difficult to know if social desirability was avoided. To minimize social desirability, future research within this field should pay attention to exploring divergent attitudes (Clark, 2011). Other studies have used follow up measures such as survey data to explore different or divergent attitudes in an anonymized setting (Sparkes & Smith, 2013).

The representativeness of the study is also important to address. We chose to include well-equipped school playgrounds with a variety of different play equipment pieces for tweens. Therefore, future studies may wish to examine this topic among tweens with limited play equipment. Our findings revealed that even with these well-equipped school playgrounds, tweens expressed a lack of play opportunities. This finding further emphasizes the need to consider how school playgrounds can support the needs of tweens.

Finally, a strength of the present study was the use of a strong theoretical framework. Previous research has found that studies guided by the SDT show strong results (Hancox et al., 2018; Sebire et al., 2013). A study of the psychometric properties of the SDT has shown that children’s motivation for PA is associated with their objectively measured PA (Sebire et al., 2013). That study argued that when assessing motivational factors of PA using SDT, researchers’ conclusions are highly reliable. As mentioned in the introduction, different theoretical frameworks focus on different aspects of health behaviour. The SDT offers an understanding of the individual motivation. Other studies of motivation have used the Achievement Goal Theory (AGT; Murphy & Alexander, 2000; Nicholls, 1989). The AGT focusses on achievement behaviour which is seen when individuals seek to develop or demonstrate high abilities (Nicholls, 1984). This conceptualization of motivation implies that people aim to avoid failure and desire achieving success through high ability. In our study, we found that tweens needed challenges and sought to practice and develop new skills. Interpreting our results using the AGT could enhance the understanding of the need for competence.

To extend the analysis of health behaviour, future studies should address other factors, not limited to the individual level, that might influence health behaviour. Applying a focus on social and structural factors influencing tweens’ health behaviour can contribute further to the understanding of tweens’ use of school playgrounds (Bronfenbrenner, 1994).

## Conclusion

In this study, we used the SDT to understand how school playgrounds can provide need support of tweens thus enhancing their autonomous motivation to use the playground. Our findings revealed several factors that either supported or thwarted the three basic psychological needs; autonomy, competence, and relatedness. Satisfaction of autonomy was generally high on school playgrounds as tweens experienced an ability to choose their play activities freely. Autonomy was supported when a variety of play equipment pieces were available and when the play equipment was placed close to the tweens’ classrooms. Autonomy was thwarted if there was insufficient play space on the playground and on the play equipment. Competence was supported by play opportunities that were challenging, tall, difficult, fast, risky, and allowed for competition and skill development. Finally, relatedness was supported by play equipment that facilitated social interaction and places to hang out. Positive experiences from social play transferred positive emotions in the classroom. These findings provide important considerations on how school playgrounds can provide need support of tweens thus resulting in higher autonomous motivation for playground use. To enhance autonomous motivation of tweens on school playgrounds, playground designers should consider the perspectives of tweens when designing school playgrounds. The conclusions of this study point to the possibility of supporting tweens in their development of autonomous motivation for using playgrounds. Focusing on tweens as a specific target group for playground design can help them to be more physically and socially active in school playgrounds. Hereby, school playgrounds can serve an important role in the fight against physical inactivity and help replace sedentary and screen-based time with social and active play.

## References

[cit0001] Adams, S., Savahl, S., & Fattore, T. (2017). Children’s representations of nature using photovoice and community mapping: Perspectives from South Africa. *International Journal of Qualitative Studies on Health and Well-Being*, 12(1), 1333900. 10.1080/17482631.2017.1333900 28699852 PMC5510192

[cit0002] Adler, K., Salanterä, S., & Zumstein-Shaha, M. (2019). Focus group interviews in child, youth, and parent research: An integrative literature review. *International Journal of Qualitative Methods*, 18, 1609406919887274. 10.1177/1609406919887274

[cit0003] Agar, M. (1996). *The professional stranger: An informal introduction to ethnography 1996 San Diego*. CA Academic Press.

[cit0004] Amholt, T. T., Jespersen, J. F., Zacho, M., Timperio, A., & Schipperijn, J. (in review). Where are tweens active in school playgrounds? A hot-spot analysis using GPS, accelerometer, and GIS data

[cit0005] Amholt, T. T., Pawlowski, C. S., Jespersen, J. F., & Schipperijn, J. (2022). Investigating the use of playgrounds by tweens: A systematic observation study. International Journal of Play.

[cit0006] Andersen, H. B., Christiansen, L. B., Pawlowski, C. S., & Schipperijn, J. (2019). What we build makes a difference – Mapping activating schoolyard features after renewal using GIS, GPS and accelerometers. *Landscape and Urban Planning*, 191, 103617. 10.1016/j.landurbplan.2019.103617

[cit0007] Aziz, N. F., & Said, I. (2015). Outdoor environments as children’s play spaces: Playground affordances. In B. Evans, J. Horton, & T. Skelton (Eds.), *Play, recreation, health and well being* (pp. 1–12). Springer. 10.1007/978-981-4585-96-5_7-1

[cit0008] Baceviciene, M., & Jankauskiene, R. (2021). Self-determined motivation mediates the association between self-reported availability of green spaces for exercising and physical activity: An explorative study. *Sustainability*, 13(3), 1312. 10.3390/su13031312

[cit0009] Black, I. E., Menzel, N. N., & Bungum, T. J. (2015). The relationship among playground areas and physical activity levels in children. *Journal of Pediatric Health Care*, 29(2), 156–168. 10.1016/j.pedhc.2014.10.001 25454386

[cit0010] Bronfenbrenner, U. (1994). Ecological models of human development. *Readings on the Development of Children*, 2(1), 37–43.

[cit0011] Brussoni, M., Olsen, L. L., Pike, I., & Sleet, D. A. (2012). Risky play and children’s safety: Balancing priorities for optimal child development. *International Journal of Environmental Research and Public Health*, 9(9), 3134–3148. 10.3390/ijerph9093134 23202675 PMC3499858

[cit0012] Bundy, A., Engelen, L., Wyver, S., Tranter, P., Ragen, J., Bauman, A., Baur, L., Schiller, W., Simpson, J. M., Niehues, A. N., Perry, G., Jessup, G., & Naughton, G. (2017). Sydney playground project: A cluster-randomized trial to increase physical activity, play, and social skills. *The Journal of School Health*, 87(10), 751–759. 10.1111/josh.12550 28876473

[cit0013] Christiansen, L. B., Toftager, M., Pawlowski, C. S., Andersen, H. B., Ersbøll, A. K., & Troelsen, J. (2017). Schoolyard upgrade in a randomized controlled study design—How are school interventions associated with adolescents’ perception of opportunities and recess physical activity. *Health Education Research*, 32(1) , cyw058. 10.1093/her/cyw058 PMC5914349 28115424

[cit0014] Clark, L. (2009). Focus group research with children and youth. *Journal for Specialists in Pediatric Nursing*, 14(2), 152–154. 10.1111/j.1744-6155.2009.00187.x 19356209

[cit0015] Clark, C. D. (2011). *In a younger voice: Doing child-centered qualitative research*. Oxford University Press.

[cit0016] Cohen, D. A., Han, B., Williamson, S., Nagel, C., McKenzie, T. L., Evenson, K. R., & Harnik, P. (2020). Playground features and physical activity in U.S. neighborhood parks. *Preventive Medicine*, 131, 105945. 10.1016/j.ypmed.2019.105945 31805315 PMC7405885

[cit0017] Cooper, C., Sorensen, W., & Yarbrough, S. (2017). Visualising the health of communities: using photovoice as a pedagogical tool in the college classroom. *Health Education Journal*, 76(4), 454–466. 10.1177/0017896917691790

[cit0018] Czalczynska-Podolska, M. (2014). The impact of playground spatial features on children’s play and activity forms: An evaluation of contemporary playgrounds’ play and social value. *Journal of Environmental Psychology*, 38, 132–142. 10.1016/j.jenvp.2014.01.006

[cit0019] Daley, A. M. (2013). Adolescent-friendly remedies for the challenges of focus group research. *Western Journal of Nursing Research*, 35(8), 1043–1059. 10.1177/0193945913483881 23618823

[cit0020] Darbyshire, P., MacDougall, C., & Schiller, W. (2005). Multiple methods in qualitative research with children: More insight or just more? *Qualitative Research*, 5(4), 417–436. 10.1177/1468794105056921

[cit0021] Deci, E. L., & Ryan, R. M. (2004). *Handbook of self-determination research*. University Rochester Press.

[cit0022] Deci, E. L., & Ryan, R. M. (2017). Self-determination theory. Adams, Nicole, Little, Todd D., Ryan, Richard. In *Development of self-determination through the life-course* (pp. 47–54). Springer Netherlands. 10.1007/978-94-024-1042-6_4

[cit0023] Dyment, J., & O’Connell, T. S. (2013). The impact of playground design on play choices and behaviors of pre-school children. *Children’s Geographies*, 11(3), 263–280. 10.1080/14733285.2013.812272

[cit0024] Escriva-Boulley, G., Tessier, D., Ntoumanis, N., & Sarrazin, P. (2018). Need-supportive professional development in elementary school physical education: Effects of a cluster-randomized control trial on teachers’ motivating style and student physical activity. *Sport, Exercise, and Performance Psychology*, 7(2), 218–234. 10.1037/spy0000119

[cit0025] Flôres, F. S., Rodrigues, L. P., Copetti, F., Lopes, F., & Cordovil, R. (2019). Affordances for motor skill development in home, school, and sport environments: A narrative review. *Perceptual and Motor Skills*, 126(3), 366–388. 10.1177/0031512519829271 30773999

[cit0026] Frost, J. L., Wortham, S. C., & Reifel, R. S. (2012). *Play and child development* (4th ed.). Pearson.

[cit0027] Gale, N. K., Heath, G., Cameron, E., Rashid, S., & Redwood, S. (2013). Using the framework method for the analysis of qualitative data in multi-disciplinary health research. *BMC Medical Research Methodology*, 13(1), 117. 10.1186/1471-2288-13-117 24047204 PMC3848812

[cit0028] Hancox, J. E., Quested, E., Ntoumanis, N., & Thøgersen-Ntoumani, C. (2018). Putting self-determination theory into practice: Application of adaptive motivational principles in the exercise domain. *Qualitative Research in Sport, Exercise and Health*, 10(1), 75–91. 10.1080/2159676X.2017.1354059

[cit0029] Hayball, F. Z., & Pawlowski, C. S. (2018). Using participatory approaches with children to better understand their physical activity behaviour. *Health Education Journal*, 77(5), 542–554. 10.1177/0017896918759567 30166649 PMC6094501

[cit0030] Herrington, S., & Brussoni, M. (2015). Beyond physical activity: The importance of play and nature-based play spaces for children’s health and development. *Current Obesity Reports*, 4(4), 477–483. 10.1007/s13679-015-0179-2 26399254

[cit0031] Horner, S. D. (2000). Using focus group methods with middle school children. *Research in Nursing & Health*, 23(6), 510–517. 10.1002/1098-240X(200012)23:6<510::AID-NUR9>3.0.CO;2-L 11130609

[cit0032] Howell, D. C. (2016). *Fundamental statistics for the behavioral sciences*. Cengage learning.

[cit0033] Huberty, J. L., Siahpush, M., Beighle, A., Fuhrmeister, E., Silva, P., & Welk, G. (2011). Ready for recess: A pilot study to increase physical activity in elementary school children. *The Journal of School Health*, 81(5), 251–257. 10.1111/j.1746-1561.2011.00591.x 21517864

[cit0034] Islam, M. Z., Moore, R., & Cosco, N. (2016). Child-friendly, active, healthy neighborhoods: Physical characteristics and children’s time outdoors. *Environment and Behavior*, 48(5), 711–736. 10.1177/0013916514554694

[cit0035] Jones, R. A., Hinkley, T., Okely, A. D., & Salmon, J. (2013). Tracking physical activity and sedentary behavior in childhood. *American Journal of Preventive Medicine*, 44(6), 651–658. 10.1016/j.amepre.2013.03.001 23683983

[cit0036] Kreutz, A., Timperio, A., & Veitch, J. (2021). Participatory school ground design: Play behaviour and student and teacher views of a school ground post-construction. *Landscape Research*, 46(6), 860–877. 10.1080/01426397.2021.1909713

[cit0037] Krueger, R. A. (2014). *Focus groups: A practical guide for applied research*. Sage publications.

[cit0038] Kusenbach, M. (2003). Street phenomenology: The go-along as ethnographic research tool. *Ethnography*, 4(3), 455–485. 10.1077/0013.9165.18806686

[cit0039] Kyttä, M. (2002). Affordances of children’s environments in the context of cities, small towns, suburbs and rural villages in Finland and Belarus. *Journal of Environmental Psychology*, 22(1), 109–123. 10.1006/jevp.2001.0249

[cit0040] Laird, Y., Fawkner, S., & Niven, A. (2018). A grounded theory of how social support influences physical activity in adolescent girls. *International Journal of Qualitative Studies on Health and Well-Being*, 13(1), 1435099. 10.1080/17482631.2018.1435099 29405881 PMC5814762

[cit0041] Larson, L. R., Szczytko, R., Bowers, E. P., Stephens, L. E., Stevenson, K. T., & Floyd, M. F. (2019). Outdoor time, screen time, and connection to nature: Troubling trends among rural youth? *Environment and Behavior*, 51(8), 966–991. 10.1177/0013916518806686

[cit0042] Leyton, M., Batista, M., Lobato, S., Aspano, M. I., & Jiménez, R. (2017). Application of two intervention programs in order to optimize motivation and to improve eating habits in adult and elderly women. *Journal of Human Kinetics*, 59(1), 131–142. 10.1515/hukin-2017-0153 29134054 PMC5680692

[cit0043] Lightfoot, C., Cole, M., & Cole, S. (2009). *The development of children* (6th ed.). Worth Publishers.

[cit0044] Lonsdale, C., Lester, A., Owen, K. B., White, R. L., Peralta, L., Kirwan, M., Diallo, T. M. O., Maeder, A. J., Bennie, A., MacMillan, F., Kolt, G. S., Ntoumanis, N., Gore, J. M., Cerin, E., Cliff, D. P., & Lubans, D. R. (2019). An internet-supported school physical activity intervention in low socioeconomic status communities: Results from the Activity and Motivation in Physical Education (AMPED) cluster randomised controlled trial. *British Journal of Sports Medicine*, 53(6), 341–347. 10.1136/bjsports-2017-097904 28993404

[cit0045] Martínez-Andrés, M., Bartolomé-Gutiérrez, R., Rodríguez-Martín, B., Pardo-Guijarro, M. J., & Martínez-Vizcaíno, V. (2017). “Football is a boys’ game”: Children’s perceptions about barriers for physical activity during recess time. *International Journal of Qualitative Studies on Health and Well-Being*, 12(sup2), 1379338. 10.1080/17482631.2017.1379338 29039264 PMC5654015

[cit0046] Murphy, P. K., & Alexander, P. A. (2000). A motivated exploration of motivation terminology. *Contemporary Educational Psychology*, 25(1), 3–53. 10.1006/ceps.1999.1019 10620380

[cit0047] Ng, J. Y. Y., Ntoumanis, N., Thøgersen-Ntoumani, C., Deci, E. L., Ryan, R. M., Duda, J. L., & Williams, G. C. (2012). Self-determination theory applied to health contexts: A meta-analysis. *Perspectives on Psychological Science*, 7(4), 325–340. 10.1177/1745691612447309 26168470

[cit0048] Nicholls, J. G. (1984). Achievement motivation: Conceptions of ability, subjective experience, task choice, and performance. *Psychological Review*, 91(3), 328. 10.1037/0033-295X.91.3.328

[cit0049] Nicholls, J. G. (1989). *The competitive ethos and democratic education*. Harvard University Press.

[cit0050] Ntoumanis, N., Taylor, I. M., & Thøgersen-Ntoumani, C. (2012). A longitudinal examination of coach and peer motivational climates in youth sport: Implications for moral attitudes, well-being, and behavioral investment. *Developmental Psychology*, 48(1), 213–223. 10.1037/0033-295X.91.3.328 21787071

[cit0051] Ntoumanis, N., Ng, J. Y. Y., Prestwich, A., Quested, E., Hancox, J. E., Thøgersen-Ntoumani, C., Deci, E. L., Ryan, R. M., Lonsdale, C., & Williams, G. C. (2021). A meta-analysis of self-determination theory-informed intervention studies in the health domain: Effects on motivation, health behavior, physical, and psychological health. *Health Psychology Review*, 15(2), 214–244. 10.1080/17437199.2020.1718529 31983293

[cit0052] Pawlowski, C. S., Schipperijn, J., Tjørnhøj-Thomsen, T., & Troelsen, J. (2016). Giving children a voice: Exploring qualitative perspectives on factors influencing recess physical activity. *European Physical Education Review* 24 1 . 10.1177/1356336X16664748

[cit0053] Pawlowski, C. S., Schmidt, T., Nielsen, J. V., Troelsen, J., & Schipperijn, J. (2019). Will the children use it?—A RE-AIM evaluation of a local public open space intervention involving children from a deprived neighbourhood. *Evaluation and Program Planning*, 77, 101706. 10.1016/j.evalprogplan.2019.101706 31472381

[cit0054] Rauner, A., Jekauc, D., Mess, F., Schmidt, S., & Woll, A. (2015). Tracking physical activity in different settings from late childhood to early adulthood in Germany: The MoMo longitudinal study. *BMC Public Health*, 15(1), 391. 10.1186/s12889-015-1731-4 25887314 PMC4407713

[cit0055] Ridgers, N. D., Stratton, G., & Fairclough, S. J. (2006). Physical activity levels of children during school playtime. *Sports Medicine*, 36(4), 359–371. 10.2165/00007256-200636040-00005 16573359

[cit0056] Roberts, H., McEachan, R., Margary, T., Conner, M., & Kellar, I. (2018). Identifying effective behavior change techniques in built environment interventions to increase use of green space: A systematic review. *Environment and Behavior*, 50(1), 28–55. 10.1177/0013916516681391

[cit0057] Ross, S. E. T., & Francis, L. A. (2016). Physical activity perceptions, context, barriers, and facilitators from a Hispanic child’s perspective. *International Journal of Qualitative Studies on Health and Well-Being*, 11(1), 31949. 10.3402/qhw.v11.31949 27534946 PMC4989179

[cit0058] Ryba, T. V., Haapanen, S., Mosek, S., & Ng, K. (2012). Towards a conceptual understanding of acute cultural adaptation: A preliminary examination of ACA in female swimming. *Qualitative Research in Sport, Exercise and Health*, 4(1), 80–97. 10.1080/2159676X.2011.653498

[cit0059] Sebire, S. J., Jago, R., Fox, K. R., Edwards, M. J., & Thompson, J. L. (2013). Testing a self-determination theory model of children’s physical activity motivation: A cross-sectional study. *International Journal of Behavioral Nutrition and Physical Activity*, 10(1), 111. 10.1186/1479-5868-10-111 24067078 PMC3852537

[cit0060] Sparkes, A. C., & Smith, B. (2013). *Qualitative research methods in sport, exercise and health: From process to product*. Routledge. 10.4324/9780203852187

[cit0061] Spencer, R. A., Numer, M., Rehman, L., & Kirk, S. F. L. (2021). Picture perfect? Gazing into girls’ health, physical activity, and nutrition through photovoice. *International Journal of Qualitative Studies on Health and Well-Being*, 16(1), 1874771. 10.1080/17482631.2021.1874771 33491602 PMC7850428

[cit0062] Sylvester, B. D., Standage, M., Dowd, A. J., Martin, L. J., Sweet, S. N., & Beauchamp, M. R. (2014). Perceived variety, psychological needs satisfaction and exercise-related well-being. *Psychology & Health*, 29(9), 1044–1061. 10.1080/08870446.2014.907900 24669787

[cit0063] Tay, G. W. N., Chan, M. J., Kembhavi, G., Lim, J., Rebello, S. A., Ng, H., Lin, C., Shek, L. P., Lança, C., Müller-Riemenschneider, F., & Chong, M.-F.-F. (2021). Children’s perceptions of factors influencing their physical activity: A focus group study on primary school children. *International Journal of Qualitative Studies on Health and Well-Being*, 16(1), 1980279. 10.1080/17482631.2021.1980279 34661503 PMC8525992

[cit0064] Telama, R., Yang, X., Viikari, J., Välimäki, I., Wanne, O., & Raitakari, O. (2005). Physical activity from childhood to adulthood: A 21-year tracking study. *American Journal of Preventive Medicine*, 28(3), 267–273. 10.1016/j.amepre.2004.12.003 15766614

[cit0065] Vansteenkiste, M., Simons, J., Lens, W., Soenens, B., & Matos, L. (2005). Examining the Motivational Impact of Intrinsic Versus Extrinsic Goal Framing and Autonomy-Supportive Versus Internally Controlling Communication Style on Early Adolescents‘ Academic Achievement. *Child Development*, 76(2), 483–501. 10.1111/j.1467-8624.2005.00858.x 15784095

[cit0066] Veitch, J., Bagley, S., Ball, K., & Salmon, J. (2006). Where do children usually play? A qualitative study of parents’ perceptions of influences on children’s active free-play. *Health & Place*, 12(4), 383–393. 10.1016/j.healthplace.2005.02.009 16814197

[cit0067] Veitch, J., Salmon, J., & Ball, K. (2007). Children’s perceptions of the use of public open spaces for active free-play. *Children’s Geographies*, 5(4), 409–422. 10.1080/14733280701631874

[cit0068] Veitch, J., Flowers, E., Ball, K., Deforche, B., & Timperio, A. (2020). Exploring children’s views on important park features: A qualitative study using walk-along interviews. *International Journal of Environmental Research and Public Health*, 17(13), 4625. 10.3390/ijerph17134625 PMC7369742 32605061

[cit0069] Veitch, J., Ball, K., Rivera, E., Loh, V., Deforche, B., & Timperio, A. (2021). Understanding children’s preference for park features that encourage physical activity: An adaptive choice based conjoint analysis. *International Journal of Behavioral Nutrition and Physical Activity*, 18(1), 133. 10.1186/s12966-021-01203-x 34627280 PMC8501594

